# Health Promotion in Practice

**Published:** 2006-09-15

**Authors:** Praphul Joshi

**Affiliations:** Health Department of Kinesiology, University of Louisiana at Lafayette, Lafayette, La

**Figure:**
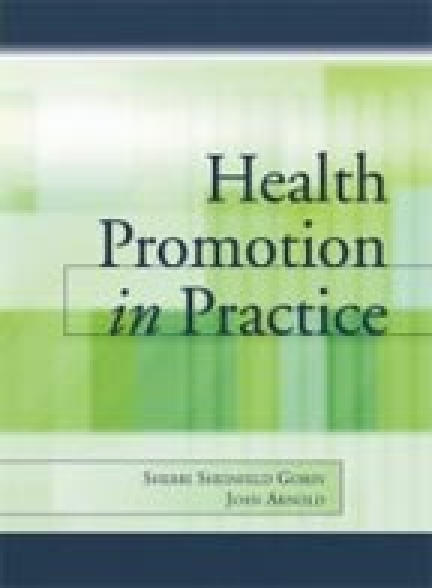


The increasing prevalence of chronic diseases has increased the global need for skilled health promotion professionals, and many professionals working in the field of health promotion lack some of the tools they need to apply in their practice. *Health Promotion in Practice* by Sherri Sheinfeld Gorin and Joan Arnold provides insight into the principles of health promotion and enables the reader to translate those principles into practice.

Various books have been published during the last two decades that have addressed health promotion theories in addition to program planning, implementation, and evaluation. *Health Promotion in Practice* not only describes the traditional theories but demonstrates how clinicians can apply these theories in practice. The authors designed this book for practicing health care professionals and advanced students from diverse health-related fields, including public health, nursing, health management, medicine, and social work.

The book contains three parts. Part One, "Health, Health Promotion, and the Health Care Professional," addresses concepts of public health, models and theories, contexts, and agents of health promotion. Chapter 2 incorporates several behavioral theories and models, as do most books on health promotion. The inclusion of Chapters 3 and 4 differentiates this book from others in the field because these chapters explain how principles of health promotion can be applied in many different types of settings.  In addition, Chapter 3 addresses the complex blend of political and economic factors that commonly affect public health practice. Since the book was written with a wide range of practitioners in mind, Chapter 4 gives valuable information on finding one's role in the field of health promotion and emphasizes the importance of collaborating and developing partnerships.

Part Two, "Practice Frameworks for Health Promotion," is the most substantial portion of the book and discusses the areas of practice — including nutrition, physical activity, sexual health, oral health, substance abuse, and injury and violence prevention — in which basic health promotion principles can be applied. Throughout this part of the book the authors have incorporated key points to be considered, and they maintain focus by addressing only topics pertinent to chronic disease prevention and health promotion. Part Three, "Economic Applications and Forecasting the Future of Health Promotion," highlights the economic future of health promotion. For example, instruction on how to evaluate finances and stay within a budget while planning and implementing programs is invaluable to professionals.

As with any book, *Health Promotion in Practice* has certain limitations. Though the concepts of evaluation are mentioned briefly, more detail on practical applications, particularly regarding process evaluation, would have been helpful. The addition of appendices (e.g., sample curricula, questionnaires and instruments, examples of mini grants, budget sheets, case studies) would be beneficial because most readers will be looking for tools to apply in the field.

Considering the intended audience and the need for a book that provides a comprehensive view of health promotion, *Health Promotion in Practice* is a great asset to the field of public health. With some additions to future editions, this book can serve as a practical and useful guide for health promotion professionals.

